# Evolutionary Dynamics of Type 2 Porcine Reproductive and Respiratory Syndrome Virus by Whole-Genome Analysis

**DOI:** 10.3390/v13122469

**Published:** 2021-12-09

**Authors:** Jiahui Guo, Zimin Liu, Xue Tong, Zixin Wang, Shangen Xu, Qian Chen, Junwei Zhou, Liurong Fang, Dang Wang, Shaobo Xiao

**Affiliations:** 1 State Key Laboratory of Agricultural Microbiology, College of Veterinary Medicine, Huazhong Agricultural University, Wuhan 430070, China; guojiahui@webmail.hzau.edu.cn (J.G.); lzm18351761871@163.com (Z.L.); tx018226@163.com (X.T.); 18568285031@163.com (Z.W.); xushangen405@163.com (S.X.); chqian0103@163.com (Q.C.); 15271914472@163.com (J.Z.); fanglr@mail.hzau.edu.cn (L.F.); vet@mail.hzau.edu.cn (S.X.); 2 Key Laboratory of Preventive Veterinary Medicine in Hubei Province, The Cooperative Innovation Center for Sustainable Pig Production, Wuhan 430070, China

**Keywords:** porcine reproductive and respiratory syndrome virus, Bayesian evolution, lineages, molecular epidemiology, recombination

## Abstract

Porcine reproductive and respiratory syndrome virus (PRRSV), an important pathogen in the swine industry, is a genetically highly diverse RNA virus. However, the phylogenetic and genomic recombination properties of this virus are not yet fully understood. In this study, we performed an integrated analysis of all available whole-genome sequences of type 2 PRRSV (n = 901) to reveal its evolutionary dynamics. The results showed that there were three distinct phylogenetic lineages of PRRSV in their distribution patterns. We identified that sublineage 2.7 (L2.7), associated with a NADC30 cluster, had the highest substitution rate and higher viral genetic diversity, and inter-lineage recombination is observed more frequently in L2.7 PRRSV compared to other sublineages. Most inter-lineage recombination events detected are observed between L2.7 PRRSVs (as major parents) and L3.4 (a JXA1-R-related cluster)/L3.7 (a WUH3-related cluster) PRRSVs (as minor parents). Moreover, the recombination hotspots are located in the structural protein gene ORF2 and ORF4, or in the non-structural protein gene nsp7. In addition, a GM2-related cluster, L3.2, shows inconsistent recombination modes compared to those of L2.7, suggesting that it may have undergone extensive and unique recombination in their evolutionary history. We also identified several amino acids under positive selection in GP2, GP4 and GP5, the major glycoproteins of PRRSV, showing the driving force behind adaptive evolution. Taken together, our results provide new insights into the evolutionary dynamics of PPRSV that contribute to our understanding of the critical factors involved in its evolution and guide future efforts to develop effective preventive measures against PRRSV.

## 1. Introduction

Porcine reproductive and respiratory syndrome (PRRS) is a viral disease characterized by two overlapping clinical presentations, complications with reproduction and respiratory disease in pigs of any age [1,2]. This disease has brought enormous economic losses to swine production in recent years, particularly following the emergence of the highly pathogenic PRRS virus (HP-PRRSV) in China and has become an intractable problem for the development of the swine industry [3,4,5]. PRRSV belongs to the genus Arterivirus and has a positive-sense RNA virus with a full-length genome of approximately 15 kb, which is organized into at least ten overlapping open reading frames (ORFs): ORF1a, ORF1b, ORF2a, ORF2b, ORF3, ORF4, ORF5a, and ORF5–ORF7, whereas ORF2a, ORF2b, ORF3, ORF4, and ORFs 5–7 encode the viral structural proteins GP2, E, GP3, GP4, GP5, M, and N, respectively [6]. ORF1a and ORF1b constitute 75% of the viral genome, encoding at least 16 mature non-structural proteins (nsp1a, nsp1b, nsp2–6, nsp2TF, nsp2N, nsp7α, nsp7β and nsp8–12) [7,8,9,10]. ORF5, encoding GP5, is one of the most variable regions of structural proteins in the PRRSV genome and is thus often used for phylogenetic analysis [11,12]. GP5 is the primary envelope protein, a glycoprotein of approximately 200 amino acids with an apparent molecular mass of 26 kDa [11]. There are 2–4 glycosylation sites, an N-terminal signal peptide directing protein synthesis to the rough endoplasmic reticulum (ER), and some antigenic determinants that induce neutralizing antibodies included in GP5 [13,14]. The presence of a major neutralization epitope in the N-terminal ectodomain implied GP5 is involved in receptor recognition [15]. Thus, GP5 is important for understanding the genetic relatedness and epidemiological status of PRRSV field isolates, and for advancing vaccine development [12,13,15].

The PRRS disease was first discovered in swine farms in the United States and Europe in the 1980s [16], with reports of its occurrence in China as early as 1996 [17]. With the emergence of HP-PRRSV strains; however, serious disease epidemics have occurred in China since 2006, and it has spread throughout the country with considerable genetic variation [5,18,19,20]. PRRSV is a rapidly evolving virus, with over 900 whole-genomes of PRRSV issued in the last years. Thus, the two genotypes of PRRSV, European type (Type 1), and North American type (Type 2), share only about 60% nucleotide similarities at the genomic level and characterized by Lelystad virus and VR-2332, respectively [16,21]. One of the key questions on PRRSV arises from its genetic diversity, which is thought to have a direct impact on immunobiology, epidemiology, diagnosis, and vaccine efficacy [22]. The widespread epidemic, persistent recurrences, and significant losses because of this intractable disease call for surveillance on the disease. Constant surveillance on PRRSV occurrence is crucial to a better understanding of the epidemiological and evolutionary dynamics of co-circulating viral lineages [9,23]. Previous studies described a comprehensive picture of the diversity of type 2 PRRSVs and classified PRRSV sequences into nine lineages based on phylogenetic analyses of ORF5 sequences [12]. However, the evolution of complete PRRSV genomes is not fully established, and there is limited knowledge about how PRRSV lineage (especially regarding vaccine-related PRRSV lineage) influences the evolution of PRRSV. In the study, we performed a comprehensive analysis of PRRSV through phylogenetic analysis, Bayesian evolution analysis, recombinant analyses, and selective pressure analyses, which will help continually update our knowledge on circulating PRRSV. Understanding viral dynamics on a regional scale could provide important insights into the local evolutionary and ecological dynamics of PRRSV and aid in developing strategies that might diminish disease impact. Meanwhile, effective and safe vaccines should be developed and selected for the prevention and control of PRRS, considering the extensive genetic diversity of PRRSV.

## 2. Materials and Methods

### 2.1. Phylogenetic and Sequence Distance Analyses 

The sequences of 75 type 1 PRRSV and 901 type 2 PRRSV were obtained from the National Center for Biotechnology Information (NCBI, https://www.ncbi.nlm.nih.gov, accessed on 1 November 2021) in August 2020 and used for analyzing phylogenetic and new lineage classification. These sequences consist of PRRSV field samples worldwide (e.g., China, the United States, and South Korea), live attenuated vaccine strains and laboratory attenuated strain. All information of sequences (GenBank ID; reported time; country; host) is classified in Table S1. A total of 976 genomic sequences were aligned using MAFFT [24]. Conserved sequence was selected with Gblocks using default parameters [25]. Multiple sequence alignments of the 976 PRRSV genomes were performed by using the genome sequence of Lactate dehydrogenase-elevating virus (GenBank accession no. NC001639) as an outgroup. To further identify the lineage classifications for all type 2 PRRSV strains circulating in the world, a complete genome phylogeny was reconstructed using IQ-TREE software. ML phylogenetic trees were constructed using IQ-TREE [26], and the best-fitting nucleotide substitution model was determined automatically by the program following 1000 bootstrap replicates. The results were visualized using iTOL v.4 (http://itol.embl.de/ (accessed on 1 November 2021)) [27]. The p-distance analyses were assessed by MEGA X [28].

### 2.2. BEAST Analyses

Bayesian molecular clock phylogenetic analysis was performed by using BEAST 1.8 [29] and then processed through recombination detection using RDP v.4.96 [30]. Sequences suspected to be recombinants were removed. To ensure that these had sufficient temporal structure in alignment for reliable rate estimation, we first performed a regression of root-to-tip genetic distances on the ML tree against exact sampling dates using the TempEst [31], and anomalous sequences were removed based on the estimated root-to-tip distance. For each lineage, two independent runs of 100 million generations were performed, sampling every 10,000 generations and removing the first 15% as burn-in. Using BEAST, time-stamped data were analyzed under the uncorrelated lognormal relaxed molecular clock [32], the GTR nucleotide substitution model [33]. The Bayesian skyline coalescent tree prior was used. Convergence was assessed using TRACER 1.7 [34].

### 2.3. Recombinant Analyses

Potential recombinations within the whole genome sequences were screened using seven methods (RDP, GENECONV, MaxChi, Bootscan, Chimera, SiScan, and 3Seq) implemented in the Recombination Detection Program version 4 (RDP4) and set *p* < 0.001 to avoid inaccurate recombination events [30]. The breakpoints were also defined by RDP4, and recombination events were indicated when four of seven methods reported recombination signals. The recombination frequency for each gene was the percentage of the recombination events that occurred at genes from the total length of genes. Recombination events with only sufficient evidence (not partial or trace evidence in the same event) and both parental strains unambiguously identified (i.e., no missing parental strain scenario) were kept. The heat map was plotted using TBtools [35].

### 2.4. Selective Pressure

For selection pressure analysis, four nucleotide sequence datasets were constructed, corresponding to ORF2, ORF4 and ORF5 nucleotide sequences of L2.7, L3.3, L3.4 and L3.7. Selection pressure (positive or purifying) was estimated for codons across the ORF2, ORF4 and ORF5 by calculating variability in the ratio of non-synonymous mutations (dN) to synonymous (dS) mutations per codon site using EasyCodeML v1.21 [36]. Likelihood ratio tests (LRTs) were performed to compare three sets of nested site-specific models, namely M3 (discrete) vs. M0 (one-ratio), M2a (positive selection) vs. M1a (nearly neutral), M8 (β & ω) vs. M7 (β) and M8 (β & ω) vs. M8a, via the CODEML algorithm [37], implemented in EasyCodeML v1.21 [36]. When LRTs yielded significant results, the Bayes Empirical Bayes (BEB) approach was used to identify codons under positive selection, i.e., positions with ω > 1 and PP > 0.95. Nucleotide sequence datasets were aligned using Align Codons in MEGA X [28].

## 3. Results

### 3.1. Phylogenetic Analyses of the Whole-Genome of PRRSV Strains

**Figure 1:**
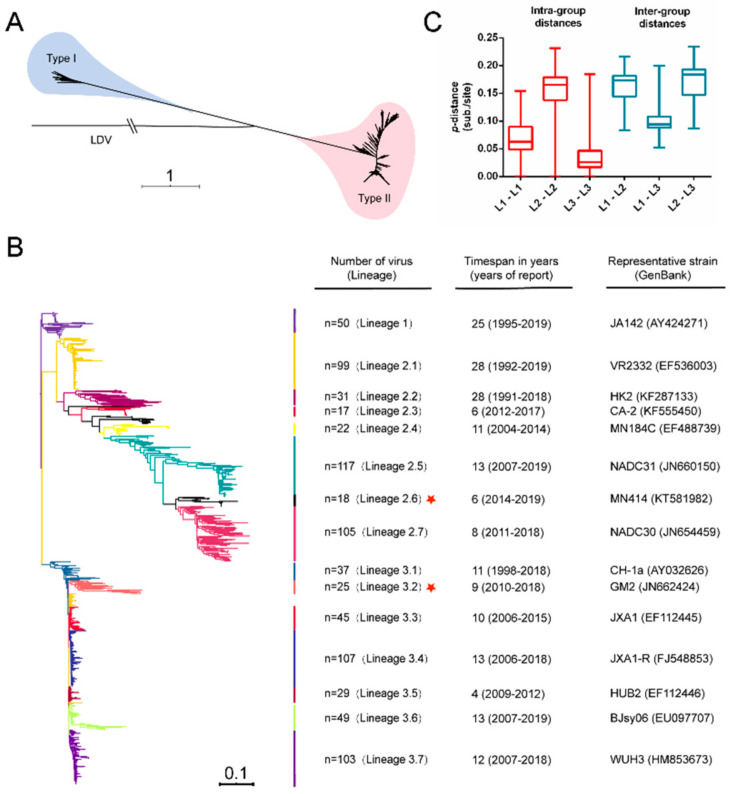
Phylogenetic and sequence distance analyses of the PRRSV (**A**) Maximum-likelihood phylogenetic tree of PRRSV genome and LDV as an outgroup, colored according to the gene type, type 1 PRRSV are shown in blue, and type 2 PRRSV are shown in pink. (**B**) Maximum-likelihood phylogenetic tree analyses of type 2 PRRSV, the clade colors indicate lineages and sublineages. Three main genetic lineages were distinguished, lineages 2 and 3 containing further clades 2.1–2.7 and 3.1–3.7, respectively. Other information is displayed next to the phylogenetic tree (Number of the virus, timespan in years, representative strains). The red star indicates two recombination lineages. Of the total L2 and L3, n = 99, n = 31, n = 17, n = 22, n = 117, n = 18 and n = 105 were classified into L2.1 to L2.7 and n = 37, n = 25, n = 45, n = 107, n = 29, n = 49 and n = 103 were classified into L3.1 to L3.7. (**C**) The nucleotide sequence divergence (p-distance) between Type 2 PRRSV intra-group distance and inter-group distance was used for comparison.

**Figure 2:**
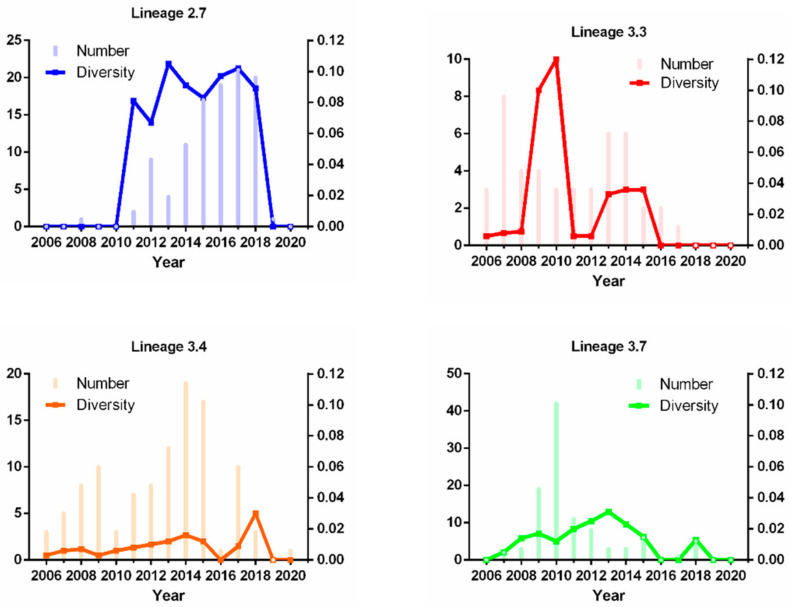
The number and diversity of PRRSV during 2006–2020 The bar chart shows the number of PRRSV sequences obtained by four sublineages during 2006–2020. The line chart shows the diversity of PRRSV sequences obtained by four sublineages during 2006–2020. The number and diversity of samples positive for PRRSV are indicated by different colored lines, respectively.

Recently, an increasing number of PRRSV whole-genome sequences have become available by next-generation sequencing (NGS) [38]. Here, we constructed a phylogenetic tree using IQ-TREE based on the 976 whole-genome sequences of PRRSV isolates, and PRRSVs can be classified into two major genotypes, type 1 (75 PRRSV isolates) and type 2 (901 PRRSV isolates) (Figure 1A), which is consistent with previous phylogenetic studies [12,16,21]. To further gain insight into type 2 PRRSV, a phylogenetic tree was constructed based on the maximum likelihood phylogenetic analysis of 901 whole-nucleotide alignments, and type 2 PRRSV isolates can be divided into three major lineages, named as lineage 1 (L1), lineage 2 (L2) and lineage 3 (L3) (Figure 1B and Figure S1). Most PRRSV strains in L2 were predominantly observed in the United States, while most PRRSV strains in L3 were reported in China. Moreover, inter-lineage strains showed considerable genetic diversity (Figure 1C), indicating a meaningful classification system. Intra-lineage strains in L2 showed greater genetic diversity than those in L1 and L3, suggesting that different evolutionary events probably influenced these three different lineages. In addition, both L2 and L3 were further divided into seven sublineages (L2.1 to L2.7; L3.1 to L3.7). Among L2 PRRSVs, L2.7 was not only predominant in the United States but also gradually became dominant in China. Another remarkable result was that a large number of sequences belonging to L3 had “abnormally” low genetic diversity. In addition, we located two important live attenuated vaccines in the overall PRRSV whole genome phylogeny. MLV vaccine strain and its parental strain, VR-2332, belonged to lineage 2.1, along with a large number of PRRSV strains in the United States. However, JXA1-R vaccine strain belonged to lineage 3.4, which contained 107 sequences with a relatively low level of diversity (Figure 2). Therefore, based on the number of strains and previous phylogenetic studies, [9,39] we selected three sublineages (L3.3, L3.4, and L3.7) of L3 and one sublineage (L2.7) of L2 for further study.

### 3.2. Intra-Sublineage Genetic Diversity and Temporal Analysis of Sublineages of Type 2 PRRSV

To investigate the genetic diversity of the major PRRSV sublineages (L2.7, L3.3, L3.4, and L3.7), we calculated the mean genetic distance of intra-sublineages and their distribution over time to illustrate the evolutionary event (Figure 2). Our analysis of the major sublineages revealed two modes of genetic diversity distribution. One of the modes of genetic diversity distribution included strains with low genetic diversity. In particular, L3.3 and L3.4 accounted for a high proportion of all sublineages and became the dominant sublineages in 2006–08 (14.15% and 15.09%), whereas L3.7 strains were reported more frequently after 2009 and occupied a larger proportion of strains (53.33%), but with lower genetic diversity. Another significant point is that L3.4 had a relatively stable number at all times, which is unusual as a vaccine-like sublineage. This result was consistent with the fact that significant HP-PRRSV outbreaks were reported in China after 2006. In contrast, L2.7, the predominant sublineage circulating in 2012–2018, showed another type of genetic diversity distribution mode, which had multiple substitutions at the nucleotide sequence, resulting in diversification into high genetic diversity. L2.7 had not only a particular temporal distribution but also a characteristic geographic distribution, showing L2.7 in both China and the United States. In general, the study of the molecular epidemiological characteristics of L2.7, L3.3, L3.4, and L3.7 will play an important role in the prevention of PRRSV.

### 3.3. Evolutionary Dynamics of L2.7, L3.3, L3.4, and L3.7 PRRSV

**Figure 3:**
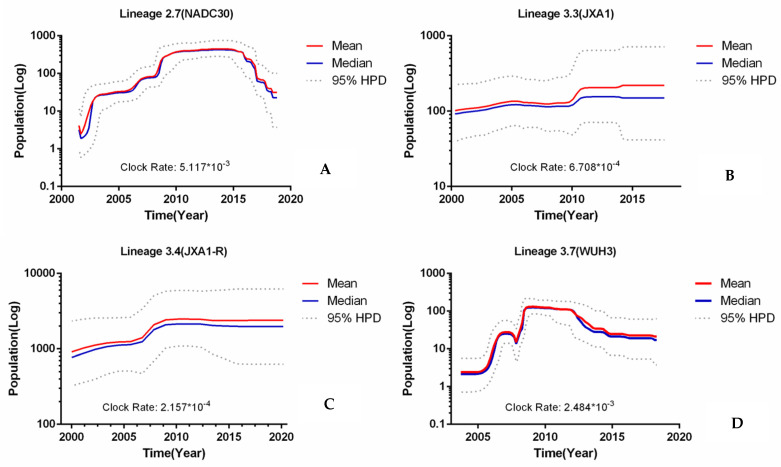
Bayesian spatiotemporal speculation of sublineages 2.7, 3.3, 3.4, and 3.7 PRRSV (**A**–**D**) Bayesian skyline population dynamic analysis. The red line indicates the mean of the size and genetic diversity of the population. The blue line indicates the median of the size and genetic diversity of the population. The dotted line indicates the 95% HPD of the size and genetic diversity of the population.

To investigate the spatiotemporal relationships of L2.7, L3.3, L3.4, and L3.7, we performed Bayesian spatiotemporal speculation using BEAST 1.8. As shown in Figure 3, the viruses in L2.7 and L3.7 evolved at the higher substitution rate, while the viruses in L3.3 and L3.3 evolved at the lower substitution rate, suggesting that the rates of substitution varied among different sublineages. For the high substitution rate mode, the BEAST estimation of L2.7 and L3.7 obtained by the strict molecular clock model yielded an average substitution rate of 5.117 × 10^−3^ and 2.484 × 10^−3^ substitutions/site/year, with a 95% credible interval of 4.8646 × 10^−3^ to 5.3617 × 10^−3^ and 2.3525 × 10^−3^ to 2.62 × 10^−3^ substitutions/site/year. The Bayesian skyline population (BSP) estimate indicated that L2.7 experienced a period of steady increase in effective population size until about 2010, which was followed by a plateau from 2010 to 2015. After 2015, a decline in population size occurred (Figure 3A), and L3.7 experienced a period of continuous increase in effective population size until about 2005, which was followed by a high plateau after 2010. After 2013, there was a further decline in population size (Figure 3D), possibly related to the control measures for HP-PRRSV. The trends of expansion and decline in population size were similar for L2.7 and L3.7. The estimation of L2.7 and L3.7 also suggested that an outbreak of NADC30-like PRRSV (L2.7) may occur soon, and effective prevention and control measures need to be taken. For the low substitution rate mode, the average substitution rate of L3.3 and L3.4 was estimated to be 6.708 × 10^−4^ and 2.157 × 10^−4^ substitutions/site/year, respectively, with a 95% credible interval of 5.6401 × 10^−4^ to 7.7855 × 10^−4^ and 1.5515 × 10^−4^ to 2.8081 × 10^−4^ substitutions/site/year. BSP estimation indicated that population size and genetic polymorphisms of L3.3 and L3.4 were in a relatively stable state during these years (2000 to 2020), which was maintained by a “plateau”; the “ plateau” in size and diversity may be related to the erroneous use of the highly pathogenic Chinese PRRSVs vaccine after 2006 (Figure 3B,C) L2.7, L3.3, L3.4, and L3.7 exhibited different population dynamics, suggesting that they experienced different evolutionary histories.

### 3.4. Positively Selective Drives Adaptive Changes of PRRSV

**Figure 4:**
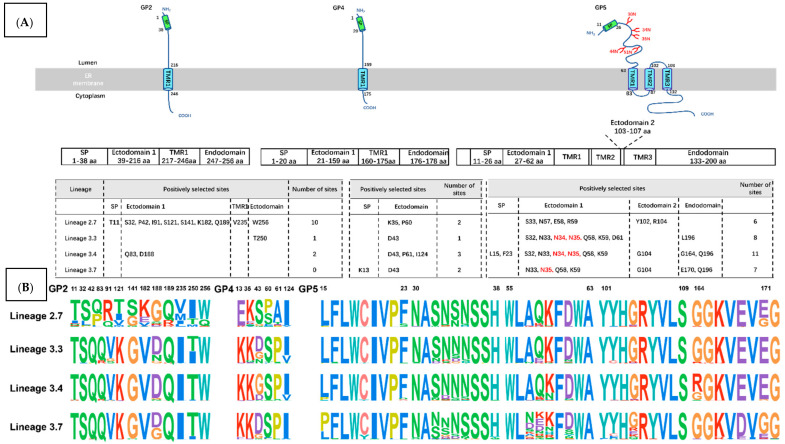
Schematic representation of PRRSV GP5 and positively selected sites. (**A**) Schematic diagram of the MEMSAT3 and MEMSATSVM predictions for PRRSV GP2, GP4 and GP5. Topology of unprocessed PRRSV GP2, GP4 and GP5 with signal peptide, ectodomain, a hydrophobic transmembrane stretch and a cytosolic/virus-internal endodomain. Red shows the location of conserved N-glycosylation sites. The lower table shows identified positively selected sites within four lineages. (**B**) Site conservation of amino acid sequences of PRRSV GP2, GP4 and GP5.

Envelope glycoproteins GP2, GP4, and GP5 are the most variable structural proteins of PRRSV and are the most viral protein that most frequently induces neutralizing antibodies [6,40,41]. Five neutralizing epitopes (41–55 and 121–135 amino acids in GP2, 40–79 amino acids in GP4, 27–31 and 37–44 amino acids in GP5) have been identified in these envelope glycoproteins [13,17,42,43,44,45]. GP5 also contains several N-glycosylation sites, two of which (N44 and N51) are highly conserved among PRRSV isolates [46,47]. The secondary structure and transmembrane helixes of GP2, GP4 and GP5 have been predicted by PRED-TMBB and PSIPRED servers [48,49]. Structurally, PRRSV GP5 (in type 2 PRRSV) has an N-terminal signal peptide (11–26 amino acids) directing protein synthesis to the rough endoplasmic reticulum (ER), passing the outer membrane three times, and has membrane-spanning strands built up-barrel structures (63–83 amino acids; 87–102 amino acids; 108–132 amino acids). The C-terminal part (132–200 amino acids) is most probably located in the cytosol and terminates inside the virus. PRRSV GP2 and GP4 (in type 2 PRRSV) have a similar structure which has an N-terminal signal peptide (1–38 amino acids; 1–20 amino acids), membrane-spanning strands (216–246 amino acids; 159–175 amino acids), and C-terminal part (247–256 amino acids; 176–178 amino acids). To investigate the possible role of GP2, GP4 and GP5 variants under selective evolutionary pressure, the ratios of non-synonymous (dN) and synonymous (dS) substitutions for ORF2, ORF4 and ORF5 among L2.7, L3.3, L3.4, and L3.7 were calculated based on the site model. Positive selection reflects viral adaptation against host immune defenses, with adaptive changes having beneficial consequences for the existence of the virus in the host. L2.7 contained 18 positively selected sites (T11, S32, P42, I91, S121, S141, K182, Q189, V235, W256 in GP2; K35, P60 in GP4; S33, N57, E58, R59, Y102, R104 in GP5); L3.3 contained 10 positively selected sites (T250 in GP2; D43 in GP4; S32, N33, N34, N35, Q58, K59, D61, L196 in GP5); L3.4 contained 16 positively selected sites (Q83, D188 in GP2; D43, P61, I124 in GP4; L15, F23, S32, N33, N34, N35, Q58, K59, G104, G164, Q196 in GP5); L3.7 contained nine positively selected sites (K13, D43 in GP4; N33, N35, Q58, K59, G104, E170, Q196 in GP5) (Figure 4). Most amino acids under selection were mainly located in the two ectodomains (26–63 amino acids;102–108 amino acids) of GP5 (Figure 4A) and these sites are highly repetitive. There are some strongly N-glycosylated sites (N34, N35). Two amino acids under selection in L3.4 (L15, F23) are located in the N-terminal signal peptide of GP5. One amino acid under selection in L3.3, L196, is located in endodomain (133–200 amino acids) of GP5. One amino acid under selection in L2.7, V235, is located in TMR1 (217–246 amino acids) of GP2, and two amino acids under selection of L3.4 (G164, Q196) and L3.7 (E170, Q196) are also located in endodomain. Comparing sequence conservation, we found that most positive selection sites have more amino acid choices. (Figure 4B). The positively selected sites for PRRSV GP2 in L2.7 were more in contrast to the L3.3, L3.4, and L3.7. These adaptive changes may have beneficial consequences for the existence of L2.7 PRRSV. However, in contrast to the L2.7, L3.3, and L3.7, the positively selected sites for PRRSV GP5 in L3.4 were higher, which means that viral variants from L3.4 may have higher fitness and are therefore preferred. Then, the result explains one of the reasons why L3.4 has a large number of strains but maintains “the low substitution rate mode”. Thus, vaccines for PRRSV require continuous monitoring and surveillance on development stage or post-market stage, which can improve the conditions of the swine industry.

### 3.5. Lineage Recombination Mode in PRRSV

**Figure 5:**
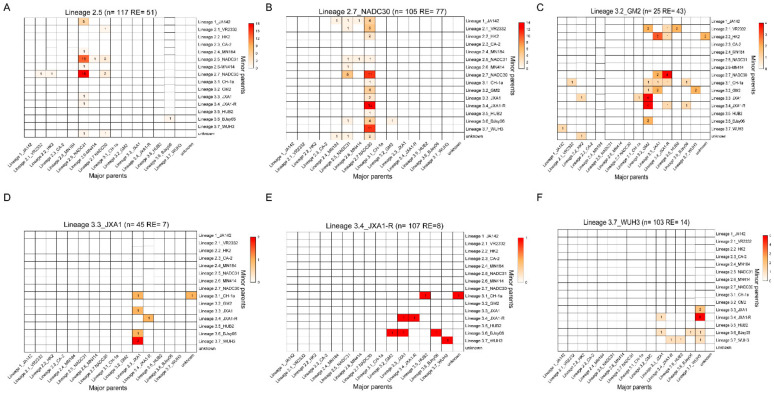
Lineage recombination mode of main sublineages (**A**–**F**) Maps of inter-lineage recombination patterns. The horizontal axis shows the 16 sublineages as major parents in Figure 5, the vertical axis shows the 16 sublineages as minor parents in Figure 5. The intensity of the color depends on the number of recombination events and the numbers in the heat map identify the number of recombination events.

Recombination has been recognized as an important process leading to genetic diversity of viral genomes upon which natural selection can function [50]. To investigate changes in the recombination modes of L1, L2, and L3 PRRSV, their recombination frequencies were first calculated from 1991 to 2019. We identified nine recombinants from 50 L1 PRRSV strains, and the recombination frequency of L1 was slightly lower than other lineages recombination at 42% (179/426) for L2 and 22% (97/425) for L3. For L2 and L3 PRRSVs, the integral recombination frequency of L2.5, L2.6, and L2.7 (approximately 43%, 77%, and 65%) was higher than that of L3.3, L3.4, and L3.7 (Figure S2). In addition, the major and minor contributions of the parental strains in the recombinants of L2 and L3 were calculated. The recombinants in L2 evolved from a complex pattern (intra- and inter- lineages) of L2 + L1, L2 + L2, and L2 + L3, whereas recombinants in L3 evolved from a singular pattern (intra-lineages) dominated by L3 + L3. Obviously, intra-lineage recombination frequencies were higher than its inter-lineage recombination for L3, especially L3.3 (intra-lineage recombination frequency at 100%), L3.4 (intra-lineage recombination frequency at 100%), and L3.7 (intra-lineage recombination frequency at 100%) (Figure 5D–F). There is a unique sublineage “L3.2” (intra-lineage recombination frequency at 65%) that differs from other sublineages (Figure 5C). The major recombination modes observed were L3 + L3 and L3 + L2, which underwent extensive recombination in the evolutionary history of L3. In contrast, for L2, intra-lineage recombination frequencies were lower than its inter-lineage recombination, especially for L2.5 and L2.7. Recombination events occurred more frequently in L2.7 than in other sublineages, and it is an important strategy for generation viral genetic diversity for L2.7 (Figure 5B). The major recombination modes observed were L2.7 + L3.4, L2.7 + L3.7, and L2.7 + L2.7 for L2.7. In addition, the recombination modes in L2.5 evolved into a singular intra-lineage pattern dominated by L2.5 + L2.5, L2.5 + L2.7 in the United States (Figure 5A). In general, the data reflected that L2.7 and L2.5 played increasingly important roles in the evolutionary history of recombination events in L2.

**Figure 6:**
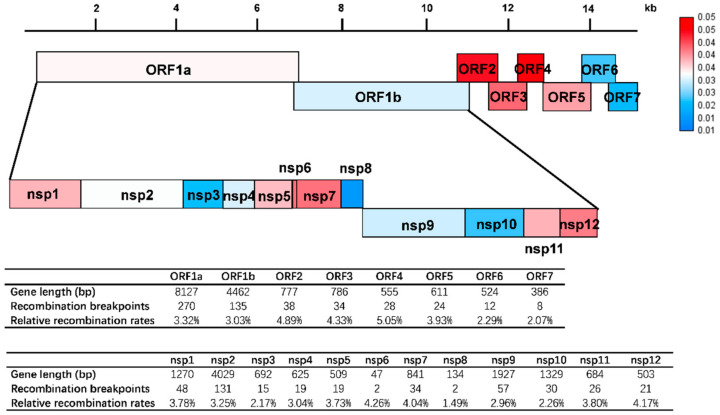
Frequency of recombination events in whole-length genome structure, regarding VR-2332, in which the positions and boundaries of the major ORFs and NSPs within ORF1a and ORF1b are shown. The intensity of the color depends on the frequency of recombination events in different ORFs and NSPs. Data are indicated below the whole-length genome structure.

Previous studies have reported the occurrence of recombination in different PRRSV strains, but the frequency of such recombination events and the genome segments involved are not well known. To map and compare the probability of recombination along the PRRSV, the relative recombination rates (the number of recombination that occurred at a gene relative to the total gene length) of each gene were calculated. The locations of recombination breakpoints were determined according to the prototype type 2 PRRSV strain. The recombination breakpoints were distributed throughout the whole-genome. In all 296 recombination events, the high-frequency recombination regions were located in nsp7 region (4.04%) which is involved in PRRSV replication, and in the GP2 and GP4 region (4.89% and 5.05%, respectively) (Figure 6). The high-frequency recombination regions of PRRSV in all recombination events genome-wide showed a similar fluctuation trend as in the L2.7 recombination events, suggesting that the recombination hotspots of PRRSV are consistent and would not change based on different lineages.

## 4. Discussion

Type 2 (or North American-like) PRRSV was first recorded in the United States in 1987, and its spread constitutes a risk for the swine industry, causing large economic losses each year [16]. Based on analyses of PRRSV GP5 sequences, previous studies described nine well-defined lineages for type 2 PRRSVs. Although the PRRSV ORF5 phylogeny has been frequently used to study genetic relationships, the full picture has hardly been revealed due to the limited information provided by PRRSV GP5. Thus, with the rapid growth of PRRSV whole genomes in databases, the phylogenetic analyses of PRRSV whole genomes needed to establish a reference sequence set for future genotyping studies. Here, we systematically investigated the genetic diversity, evolutionary dynamics, and recombination of type 2 PRRSV. The phylogenetic tree indicated that the complete PRRSV genomes were classified into three separate genogroups (L1, L2 and L3). The sequences of L2 and L3 accounted for a large proportion as the dominant lineages in different years, which is a suitable model to detect the genetic variation lineage of PRRSV. This study was the first to calculate the genetic diversity of type 2 PRRSV. Type 2 PRRSVs showed completely different genetic diversity in L2 and L3, suggesting that L2 and L3 are probably influenced by different evolutionary events. We investigated the reasons for this phenomenon, which would provide valuable information and new insights for PRRSV epidemic trends and control strategies. 

From the perspective of molecular evolutionary dynamics, L2.7, representing by the currently prevalent isolate NADC30, is undergoing a reduction in genetic diversity, which is in line with the trend in which the diversity of a certain sublineage is reduced as it becomes the dominant population before the occurrence of an epidemic. Therefore, we predict that an outbreak of L2.7 may occur in the near future. Moreover, L3.7 experienced evolutionary events similar to L2.7 and kept stable in China. Our data suggested the need to protect against related viruses that have a high substitution rate mode. We would like to predict when the new PRRSV pathogen with high substitution rate mode re-emerges, which reminds us to constantly have surveillance on some lineages (L2.7 and L3.7). In addition, although several commercial live attenuated live vaccines have been widely used in China, PRRS is still severe in the swine industry. [51,52,53] The genetic diversity and complexity of PRRSV are further increasing. Some vaccine lineages (L3.4) are currently undergoing a “high plateau” in population size and positively selected results explain it. We hope our study could not only facilitate the correct selection of vaccines but also a light on directions for future vaccine development.

Recombination is a pervasive phenomenon among PRRSV isolates and is an important strategy for generation viral genetic diversity [39,51,54]. L1, L2, and L3 were systematically screened for inter- and intra-lineage recombination. Despite our limited understanding of the reason for recombination events in type 2 PRRSV, the recombinant analyses update our understanding of the recombination model of PRRSV, allowing us to understand the frequency of such recombination events. In the analysis of recombination, we reviewed the recombination modes in type 2 PRRSV in this study and found several differences in recombination patterns between L2 and L3. Our data reflected that recombinants in L2 evolved from a complex pattern (intra- and inter-lineages) of L2 + L1, L2 + L2, and L2 + L3, whereas recombinants in L3 evolved from a singular pattern (intra-lineages) dominated by L3 + L3. In addition, L2 PRRSVs were extremely active in the recombination event, which we attributed to some PRRSV genotypes that were more susceptible to recombination, particularly L2.7 PRRSVs (Figure S2), and co-circulate was also an important factor in preventing and controlling PRRSV difficultly. The minor parents of recombinants have gradually focused on L3.4 PRRSVs and provide adaptive evolutionary gene when it currently co-circulating with other lineages, which is an important reservoir for the genetic recombination of PRRSVs. There is a unique sublineage “L3.2” (intra-lineage recombination frequency at 65%) that underwent extensive recombination in the evolutionary history of L3 and is distinct from other sublineages. 

In our analysis of recombination, we also examined recombination breakpoints in the PRRSVs sequenced in this study. Attractively, the recombination hotspots were located in the nsp7 region (4.04%), which is involved in the replication of PRRSV, and in the GP2 and GP4 region (4.89% and 5.05%).GP2 and GP4 are the main factors of PRRSV entry into cultured cells [39]. Recombination of PRRSV at these regions may be associated with an increase in replication capacity and cellular tropism, which is thought to facilitate survival and spread and ultimately drive the pathogenesis of PRRSV. As to what type of gene structure causes high recombination frequency, further exploration of this potential mechanism is needed. In short, our analysis would provide valuable information and new insights for PRRSV epidemic trends and control strategies. We hope it will help in the future prevention and control of PRRSV.

Finally, these results will help us to understand the recombination mode of PRRSV, which guides us to focus on the monitoring of the PRRSV recombinant virus. These facts prompt people to re-evaluate the strategies of prevention and control of PRRSV all over the world, and people become more cautious about massive vaccination. Maybe they can look for a new vaccine development strategy that is safe. Combining PRRSV recombination mode, positively selective, and molecular evolutionary dynamics would help with future prevention and control of PRRSV.

## Data Availability

The datasets generated for this study are available on request to the corresponding author.

## Supplementary Materials

The following are available online at https://www.mdpi.com/article/10.3390/v13122469/s1, Figure S1: Maximum-likelihood phylogenetic tree analyses of type 2 PRRSV, Figure S2: Lineage recombination mode of all sublineages (L1, L2.1-2.7 and L3.1-3.7), Table S1: All information of PRRSV whole-genome sequences downloaded from GenBank in August 2020 (GenBank ID; reported time; country; host).

## References

[1] Done S.H., Paton D.J., White M.E. (1996). Porcine reproductive and respiratory syndrome (PRRS): A review, with emphasis on pathological, virological and diagnostic aspects. Br. Vet. J..

[2] Pang Y., Li M., Zhou Y., Liu W., Tao R., Zhang H., Xiao S., Fang L. (2020). The ubiquitin proteasome system is necessary for efficient proliferation of porcine reproductive and respiratory syndrome virus. Vet. Microbiol..

[3] Neumann E.J., Kliebenstein J.B., Johnson C.D., Mabry J.W., Bush E.J., Seitzinger A.H., Green A.L., Zimmerman J.J. (2005). Assessment of the economic impact of porcine reproductive and respiratory syndrome on swine production in the United States. Javma J. Am. Vet. Med Assoc..

[4] Tian K., Yu X., Zhao T., Feng Y., Cao Z., Wang C., Hu Y., Chen X., Hu D., Tian X. (2007). Emergence of Fatal PRRSV Variants: Unparalleled Outbreaks of Atypical PRRS in China and Molecular Dissection of the Unique Hallmark. PLoS ONE.

[5] Tong G.Z., Zhou Y.J., Hao X.F., Tian Z.J., An T.Q., Qiu H.J. (2007). Highly pathogenic porcine reproductive and respiratory syndrome, china. Emerg. Infect. Dis..

[6] Johnson C.R., Griggs T.F., Gnanandarajah J., Murtaugh M.P. (2011). Novel structural protein in porcine reproductive and respiratory syndrome virus encoded by an alternative ORF5 present in all arteriviruses. J. Gen. Virol..

[7] Snijder E.J., van Tol H., Roos N., Pedersen K.W. (2001). Non-structural proteins 2 and 3 interact to modify host cell membranes during the formation of the arterivirus replication complex. J. Gen. Virol..

[8] Li Y., Tas A., Snijder E.J., Fang Y. (2012). Identification of porcine reproductive and respiratory syndrome virus ORF1a-encoded non-structural proteins in virus-infected cells. J. Gen. Virol..

[9] Sun Y.-K., Han X.-L., Wei Y.-F., Yu Z.-Q., Ji C.-H., Li Q., Lu G., Shen L., Ma C.-Q., Wang H. (2019). Phylogeography, phylodynamics and the recent outbreak of lineage 3 porcine reproductive and respiratory syndrome viruses in China. Transbound. Emerg. Dis..

[10] Fang Y., Snijder E. (2010). The PRRSV replicase: Exploring the multifunctionality of an intriguing set of nonstructural proteins. Virus Res..

[11] Nilubol D., Tripipat T., Hoonsuwan T., Tipsombatboon P., Piriyapongsa J. (2013). Genetic diversity of the ORF5 gene of porcine reproductive and respiratory syndrome virus (PRRSV) genotypes I and II in Thailand. Arch. Virol..

[12] Shi M., Lam T.T., Hon C.C., Murtaugh M.P., Davies P.R., Hui R.K., Li J., Wong L.T., Yip C.W., Jiang J.W. (2010). Phylogeny-Based Evolutionary, Demographical, and Geographical Dissection of North American Type 2 Porcine Reproductive and Respiratory Syndrome Viruses. J. Virol..

[13] Thaa B., Sinhadri B.C., Tielesch C., Krause E., Veit M. (2013). Signal Peptide Cleavage from GP5 of PRRSV: A Minor Fraction of Molecules Retains the Decoy Epitope, a Presumed Molecular Cause for Viral Persistence. PLoS ONE.

[14] Du T., Nan Y., Xiao S., Zhao Q., Zhou E.-M. (2017). Antiviral Strategies against PRRSV Infection. Trends Microbiol..

[15] Yin G., Gao L., Shu X., Yang G., Guo S., Li W. (2012). Genetic Diversity of the ORF5 Gene of Porcine Reproductive and Respiratory Syndrome Virus Isolates in Southwest China from 2007 to 2009. PLoS ONE.

[16] Collins J., Benfield D., Christianson W.T., Harris L., Hennings J.C., Shaw D.P., Goyal S.M., McCullough S., Morrison R.B., Joo H.S. (1992). Isolation of Swine Infertility and Respiratory Syndrome Virus (Isolate ATCC VR-2332) in North America and Experimental Reproduction of the Disease in Gnotobiotic Pigs. J. Vet. Diagn. Investig..

[17] Valícek L., Psikal I., Smíd B., Rodák L., Kubalíková R., Kosinová E. (1997). Isolation and identification of porcine reproductive and respiratory syndrome virus in cell cultures. Vet. Med..

[18] Guo A., Wu G., Gong W., Luo X., Zheng H., Jia H., Cai X. (2012). Outbreaks of highly pathogenic porcine reproductive and respiratory syndrome in Jiangxi province, China. Ir. Vet. J..

[19] Liu J.-K., Wei C.-H., Yang X.-Y., Hou X.-L., Dai A.-L., Li X.-H., Wei M.-K., Pan X.-Z. (2013). Genetic diversity and evolutionary characterization of Chinese porcine reproductive and respiratory syndrome viruses based on NSP2 and ORF5. Arch. Virol..

[20] Wang C., Zhao Q., Liang C., Dang L., Ma Y., Gao J., Li Q., Huang B., Kong N., Zhang C. (2012). Complete Genome Sequence of a Highly Pathogenic Porcine Reproductive and Respiratory Syndrome Virus Variant. J. Virol..

[21] Wensvoort G., Terpstra C., Pol J.M.A., Ter Laak E.A., Bloemraad M., de Kluyver E.P., Kragten C., van Buiten L., den Besten A., Wagenaar F. (1991). Mystery swine disease in the Netherlands: The isolation of Lelystad virus. Veter. Q..

[22] Martin-Valls G.E., Kvisgaard L.K., Tello M., Darwich L., Cortey M., Burgara-Estrella A.J., Hernández J., Larsen L.E., Mateu E. (2014). Analysis of ORF5 and Full-Length Genome Sequences of Porcine Reproductive and Respiratory Syndrome Virus Isolates of Genotypes 1 and 2 Retrieved Worldwide Provides Evidence that Recombination Is a Common Phenomenon and May Produce Mosaic Isolates. J. Virol..

[23] Sun Y., Chen Y., Cai Y., Li Q., Xie J., Liang G., Gao Q., Yu Z., Lu G., Huang L. (2020). Insights into the evolutionary history and epidemiological characteristics of the emerging lineage 1 porcine reproductive and respiratory syndrome viruses in China. Transbound. Emerg. Dis..

[24] Katoh K., Misawa K., Kuma K., Miyata T. (2002). MAFFT: A novel method for rapid multiple sequence alignment based on fast Fourier transform. Nucleic Acids Res..

[25] Zhang D., Gao F., Jakovlić I., Zhou H., Zhang J., Li W.X., Wang G.T. (2020). PhyloSuite: An integrated and scalable desktop platform for streamlined molecular sequence data management and evolutionary phylogenetics studies. Mol. Ecol. Resour..

[26] Nguyen L.-T., Schmidt H.A., Von Haeseler A., Minh B.Q. (2015). IQ-TREE: A Fast and Effective Stochastic Algorithm for Estimating Maximum-Likelihood Phylogenies. Mol. Biol. Evol..

[27] Letunic I., Bork P. (2007). Interactive Tree Of Life (iTOL): An online tool for phylogenetic tree display and annotation. Bioinformatics.

[28] Kumar S., Stecher G., Li M., Knyaz C., Tamura K. (2018). MEGA X: Molecular Evolutionary Genetics Analysis across Computing Platforms. Mol. Biol. Evol..

[29] Drummond A.J., Rambaut A. (2007). BEAST: Bayesian evolutionary analysis by sampling trees. BMC Evol. Biol..

[30] Martin D.P., Murrell B., Golden M., Khoosal A., Muhire B. (2015). RDP4: Detection and analysis of recombination patterns in virus genomes. Virus Evol..

[31] Rambaut A., Lam T.T., Max Carvalho L., Pybus O.G. (2016). Exploring the temporal structure of heterochronous sequences using TempEst (formerly Path-O-Gen). Virus Evol..

[32] Li W.L.S., Drummond A.J. (2012). Model Averaging and Bayes Factor Calculation of Relaxed Molecular Clocks in Bayesian Phylogenetics. Mol. Biol. Evol..

[33] Shapiro B., Rambaut A., Drummond A. (2006). Choosing Appropriate Substitution Models for the Phylogenetic Analysis of Protein-Coding Sequences. Mol. Biol. Evol..

[34] Rambaut A., Drummond A.J., Xie D., Baele G., Suchard M.A. (2018). Posterior Summarization in Bayesian Phylogenetics Using Tracer 1.7. Syst. Biol..

[35] Chen C., Chen H., Zhang Y., Thomas H.R., Frank M.H., He Y., Xia R. (2020). TBtools: An Integrative Toolkit Developed for Interactive Analyses of Big Biological Data. Mol. Plant.

[36] Gao F., Chen C., Arab D.A., Du Z., He Y., Ho S.Y.W. (2019). EasyCodeML: A visual tool for analysis of selection using CodeML. Ecol. Evol..

[37] Yang Z. (2007). PAML 4: Phylogenetic Analysis by Maximum Likelihood. Mol. Biol. Evol..

[38] Lalonde C., Provost C., Gagnon C.A. (2020). Whole-Genome Sequencing of Porcine Reproductive and Respiratory Syndrome Virus from Field Clinical Samples Improves the Genomic Surveillance of the Virus. J. Clin. Microbiol..

[39] Yu F., Yan Y., Shi M., Liu H.Z., Zhang H.L., Yang Y.B., Huang X.Y., Gauger P.C., Zhang J., Zhang Y.H. (2020). Phylogenetics, Genomic Recombination, and NSP2 Polymorphic Patterns of Porcine Reproductive and Respiratory Syndrome Virus in China and the United States in 2014–2018. J. Virol..

[40] Kwon B., Ansari I., Pattnaik A.K., Osorio F.A. (2008). Identification of virulence determinants of porcine reproductive and respiratory syndrome virus through construction of chimeric clones. Virology.

[41] Veit M., Matczuk A.K., Sinhadri B.C., Krause E., Thaa B. (2014). Membrane proteins of arterivirus particles: Structure, topology, processing and function. Virus Res..

[42] Plagemann P.G. (2004). The primary GP5 neutralization epitope of North American isolates of porcine reproductive and respiratory syndrome virus. Vet. Immunol. Immunopathol..

[43] Plagemann P.G., Rowland R.R., Faaberg K.S. (2002). The primary neutralization epitope of porcine respiratory and reproductive syndrome virus strain VR-2332 is located in the middle of the GP5 ectodomain. Arch. Virol..

[44] Meulenberg J.J., van Nieuwstadt A.P., van Essen-Zandbergen A., Langeveld J.P. (1997). Posttranslational processing and identification of a neutralization domain of the GP4 protein encoded by ORF4 of Lelystad virus. J. Virol..

[45] De Lima M., Pattnaik A.K., Flores E.F., Osorio F.A. (2006). Serologic marker candidates identified among B-cell linear epitopes of Nsp2 and structural proteins of a North American strain of porcine reproductive and respiratory syndrome virus. Virology.

[46] Ansari I.H., Kwon B., Osorio F.A., Pattnaik A.K. (2006). Influence of N-Linked Glycosylation of Porcine Reproductive and Respiratory Syndrome Virus GP5 on Virus Infectivity, Antigenicity, and Ability To Induce Neutralizing Antibodies. J. Virol..

[47] Wei Z., Lin T., Sun L., Li Y., Wang X., Gao F., Liu R., Chen C., Tong G., Yuan S. (2012). N-Linked Glycosylation of GP5 of Porcine Reproductive and Respiratory Syndrome Virus Is Critically Important for Virus ReplicationIn Vivo. J. Virol..

[48] Bagos P.G., Liakopoulos T.D., Spyropoulos I.C., Hamodrakas S.J. (2004). PRED-TMBB: A web server for predicting the topology of beta-barrel outer membrane proteins. Nucleic Acids Res..

[49] Buchan D.W.A., Minneci F., Nugent T.C.O., Bryson K., Jones D.T. (2013). Scalable web services for the PSIPRED Protein Analysis Workbench. Nucleic Acids Res..

[50] Spencer C.C., Deloukas P., Hunt S., Mullikin J., Myers S., Silverman B., Donnelly P., Bentley D., McVean G. (2006). The influence of recombination on human genetic diversity. PLoS Genet.

[51] Lu W.H., Tun H.M., Sun B.L., Mo J., Zhou Q.F., Deng Y.X., Xie Q.M., Bi Y.Z., Leung F.C., Ma J.Y. (2015). Re-emerging of porcine respiratory and reproductive syndrome virus (lineage 3) and increased pathogenicity after genomic recombination with vaccine variant. Vet. Microbiol..

[52] Oh T., Kim H., Park K.H., Jeong J., Yang S., Kang I., Chae C. (2019). Comparison of four commercial PRRSV MLV vaccines in herds with co-circulation of PRRSV-1 and PRRSV-2. Comp. Immunol. Microbiol. Infect. Dis..

[53] Murtaugh M.P., Genzow M. (2011). Immunological solutions for treatment and prevention of porcine reproductive and respiratory syndrome (PRRS). Vaccine.

[54] Franzo G., Cecchinato M., Martini M., Ceglie L., Gigli A., Drigo M. (2014). Observation of high recombination occurrence of Porcine Reproductive and Respiratory Syndrome Virus in field condition. Virus Res..

