# Supplementary figures and images for: Evolutionary Dynamics of Type 2 Porcine Reproductive and Respiratory Syndrome Virus by Whole-Genome Analysis
Source: Viruses. 2021 Dec 9;13(12):2469. doi: 10.3390/v13122469 (PMC8706008; doi:10.3390/v13122469)

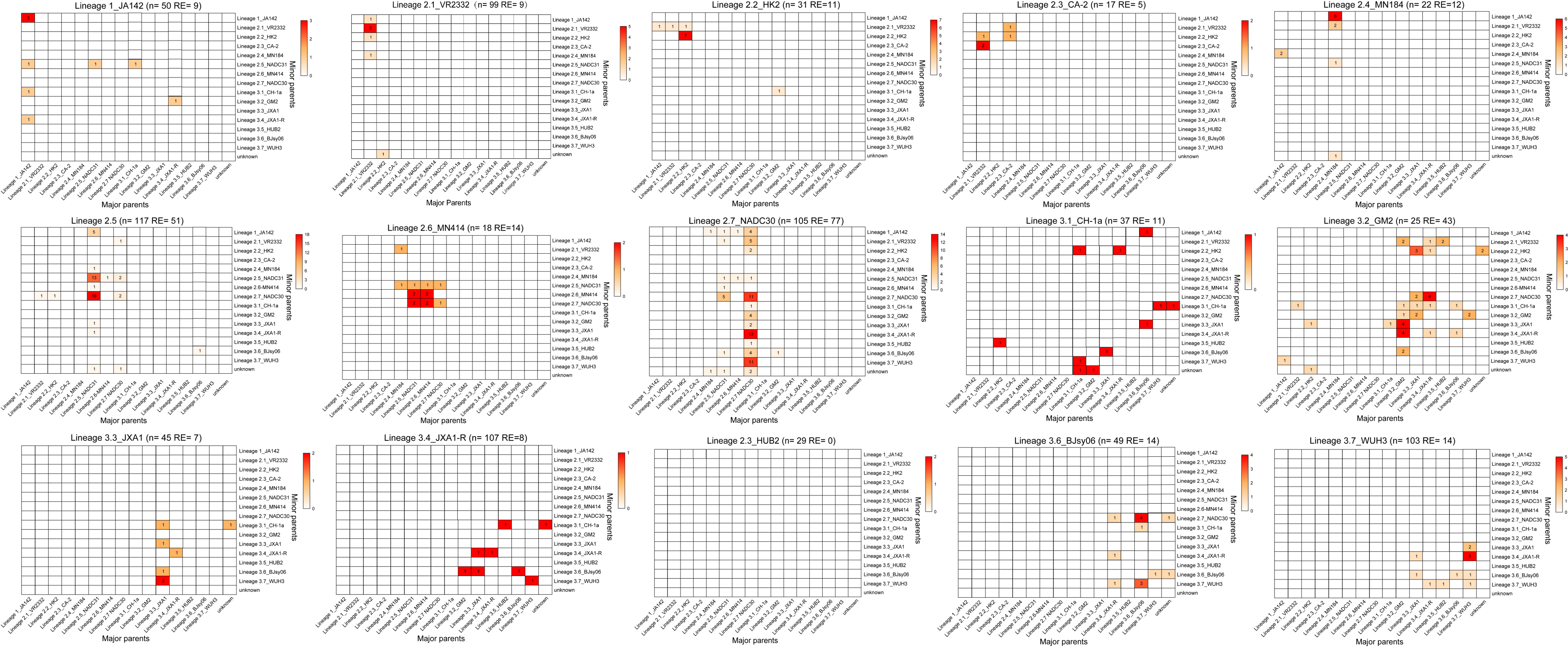

Supplement: Supplementary file 1 [file viruses-13-02469-s001.zip › Figure S2.tif]
